# Reliable assessment of telomere maintenance mechanisms in neuroblastoma

**DOI:** 10.1186/s13578-022-00896-2

**Published:** 2022-09-24

**Authors:** Alina Meeser, Christoph Bartenhagen, Lisa Werr, Anna-Maria Hellmann, Yvonne Kahlert, Nadine Hemstedt, Peter Nürnberg, Janine Altmüller, Sandra Ackermann, Barbara Hero, Thorsten Simon, Martin Peifer, Matthias Fischer, Carolina Rosswog

**Affiliations:** 1 Department of Experimental Pediatric Oncology, University Children’s Hospital of Cologne, Kerpener Str. 62, 50937 Cologne, Germany; 2 Center for Molecular Medicine Cologne, Medical Faculty, University of Cologne, Cologne, Germany; 3 Cologne Center for Genomics (CCG), University of Cologne, Faculty of Medicine and University Hospital Cologne, Cologne, Germany; 4 Core Facility Genomics, Berlin Institute of Health at Charité – Universitätsmedizin Berlin, Berlin, Germany; 5 Max Delbrück Center for Molecular Medicine in the Helmholtz Association (MDC), Berlin, Germany; 6 Department of Pediatric Oncology and Hematology, University of Cologne, Cologne, Germany; 7 Department of Translational Genomics, Center of Integrated Oncology Cologne-Bonn, Medical Faculty, University of Cologne, Cologne, Germany

**Keywords:** Neuroblastoma, Telomere maintenance, Alternative lengthening of telomeres, Telomerase

## Abstract

**Background:**

Telomere maintenance mechanisms (TMM) are a hallmark of high-risk neuroblastoma, and are conferred by activation of telomerase or alternative lengthening of telomeres (ALT). However, detection of TMM is not yet part of the clinical routine, and consensus on TMM detection, especially on ALT assessment, remains to be achieved.

**Methods:**

Whole genome sequencing (WGS) data of 68 primary neuroblastoma samples were analyzed. Telomere length was calculated from WGS data or by telomere restriction fragment analysis (n = 39). ALT was assessed by C-circle assay (CCA, n = 67) and detection of ALT-associated PML nuclear bodies (APB) by combined fluorescence in situ hybridization and immunofluorescence staining (n = 68). RNA sequencing was performed (n = 64) to determine expression of *TERT* and telomeric long non-coding RNA (TERRA). Telomerase activity was examined by telomerase repeat amplification protocol (TRAP, n = 15).

**Results:**

Tumors were considered as telomerase-positive if they harbored a *TERT* rearrangement, *MYCN* amplification or high *TERT* expression (45.6%, 31/68), and ALT-positive if they were positive for APB and CCA (19.1%, 13/68). If all these markers were absent, tumors were considered TMM-negative (25.0%, 17/68). According to these criteria, the majority of samples were classified unambiguously (89.7%, 61/68). Assessment of additional ALT-associated parameters clarified the TMM status of the remaining seven cases with high likelihood: ALT-positive tumors had higher TERRA expression, longer telomeres, more telomere insertions, a characteristic pattern of telomere variant repeats, and were associated with *ATRX* mutations.

**Conclusions:**

We here propose a workflow to reliably detect TMM in neuroblastoma. We show that unambiguous classification is feasible following a stepwise approach that determines both, activation of telomerase and ALT. The workflow proposed in this study can be used in clinical routine and provides a framework to systematically and reliably determine telomere maintenance mechanisms for risk stratification and treatment allocation of neuroblastoma patients.

**Supplementary Information:**

The online version contains supplementary material available at 10.1186/s13578-022-00896-2.

## Background

Neuroblastoma is the most common extracranial solid cancer in childhood and arises from the developing sympathetic nervous system [1, 2]. The clinical courses of neuroblastoma are diverse: Patients with low-risk neuroblastoma often show spontaneous regression, while high-risk patients die in about 50% from the disease despite multimodal aggressive treatment [3]. Current risk stratification of neuroblastoma patients is primarily based on clinical variables and molecular markers, such as genomic amplification of *MYCN* [4]*.* While risk assessment of neuroblastoma patients has been continuously improved over the last few decades, there are still subgroups of patients that may be misclassified by current strategies [5].

**Fig. 1:**
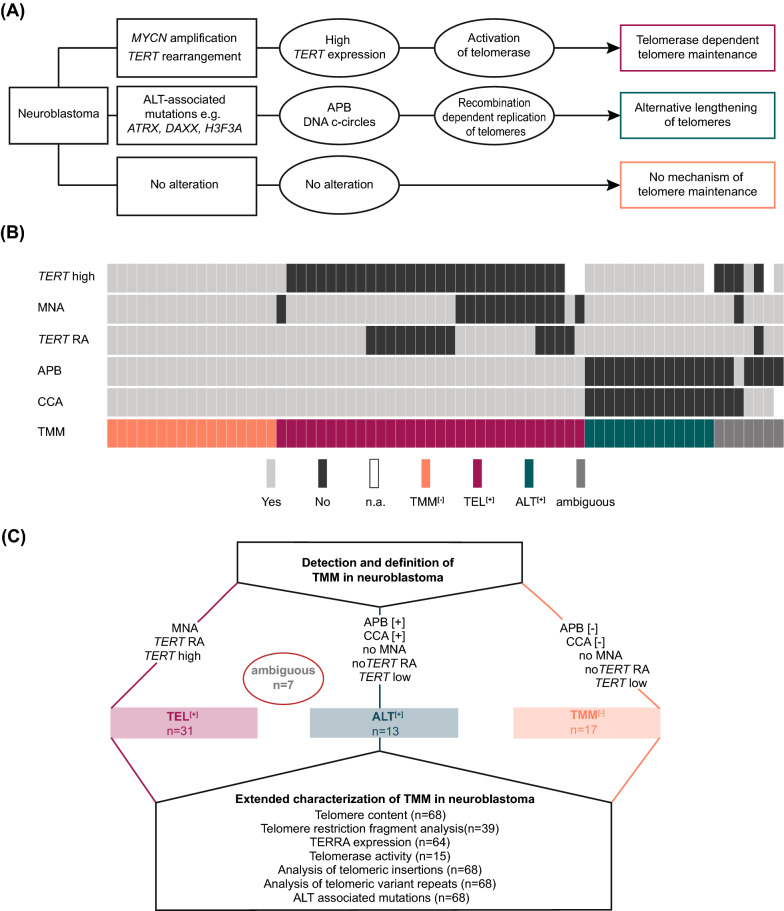
Defining telomere maintenance subgroups in neuroblastoma. **A** Neuroblastoma subgroups defined by telomere maintenance mechanisms. Genomic alterations and biomarkers associated with telomerase and ALT activation in neuroblastoma are indicated. **B** TMM characteristics and classification of neuroblastomas of the study cohort. **C** Definition of telomere maintenance subgroups in this study. Ambiguous, samples that could not unambiguously be classified by the predefined criteria

We have recently shown that the activation of telomere maintenance mechanisms (TMM) is a hallmark of high-risk neuroblastoma, while such mechanisms are invariably lacking in low-risk tumors [6]. Stabilization of the telomeres is an essential prerequisite for cancer cells to gain unlimited replicative capacity [7, 8]. Telomere maintenance may be mediated by a telomerase dependent or a telomerase independent pathway (Fig. 1A) [6, 9]. The telomerase dependent pathway is activated through transcriptional upregulation of the gene telomerase reverse transcriptase (*TERT*) which is most frequently caused by amplification of the oncogene *MYCN* or by rearrangements of the *TERT* locus in neuroblastoma [10]. The telomerase independent pathway, termed alternative lengthening of telomeres (ALT), comprises homologous recombination dependent replication of telomeres [11], but has not been completely elucidated on the molecular level so far. There are, however, several molecular alterations and parameters associated with an ALT phenotype, such as mutations in the genes *ATRX*, *DAXX*, *H3F3A* or *SMARCAL1* (Fig. 1A) [6, 12, 13].

The mechanistic classification of neuroblastoma based on TMM has been suggested as a diagnostic tool to accurately predict the natural course of disease of neuroblastoma in clinical risk estimation systems. In addition, ALT-associated alterations and telomerase may offer specific therapeutic targets [13–15]. However, no consensus strategy for assessing TMM in neuroblastoma, especially for ALT, has been established yet. Telomerase-mediated TMM in neuroblastoma can be determined by detecting the respective genomic alterations, i.e., amplification of *MYCN* (MNA) or rearrangements of *TERT* (*TERT* RA) [10], or by examining *TERT* expression levels [6, 9, 10] or telomerase activity [6, 12]. Assessment of ALT has remained more challenging, as ALT-related molecular markers may lack sensitivity or specificity [9, 12, 16–20]. Both analysis of ALT-associated promyelocytic leukemia nuclear bodies (APB) and detection of extrachromosomal circular partially double-stranded telomeric DNA (C-circles) have been used to assess ALT in neuroblastoma, however, the accuracy and comparability of these two methods have not been systematically determined yet. In addition, it is not clear, still, which additional parameters may help to determine ALT in neuroblastoma in cases with conflicting results.

We here set out to develop a diagnostic workflow to reliably determine TMM in neuroblastoma in clinical practice by comprehensively characterizing features of telomere maintenance in a cohort of neuroblastoma samples covering the entire spectrum of the disease.

## Results

To reliably determine telomerase and ALT-dependent TMM in neuroblastoma, we comprehensively examined TMM-related features in a cohort of 68 neuroblastoma samples, covering the entire spectrum of the disease (Additional file 1:Table S1). In a first step, we examined genomic alterations associated with induction of telomerase, i.e., *TERT* RA and MNA, as well as *TERT* mRNA expression levels. In addition, we assessed whether ALT was activated in the tumors by analysis of C-circles (CCA) and APB.

### Assessment of telomerase-mediated telomere maintenance

Telomerase-mediated TMM was determined by detection of MNA or *TERT* RA using FISH, and by analysis of *TERT* RNA expression levels using RNA sequencing (Additional file 2: Table S2). Based on RNA sequencing data of a larger neuroblastoma cohort [21], a threshold for high *versus* low *TERT* expression was defined (Additional file 4: Fig. S1A) according to the definition of a threshold that had been used previously [6]. Application of the threshold to the study cohort revealed elevated *TERT* expression in 50.0% of the cases (32/64) (Fig. 1B). MNA and *TERT* RA occurred in 20.6% of the tumors (14/68) each, with co-occurrence of these alterations in three cases. The latter cases were excluded from statistical comparisons between MNA and *TERT* RA tumors. All tumors bearing MNA or *TERT* RA had elevated *TERT* expression levels, except one case with MNA (mean log_2_ *TERT* expression score: *TERT* RA (n = 9), 12.1; MNA (n = 9), 9.0; other (n = 25), 7.1; ALT (n = 12), 6.0; Additional file 4: Fig. S1B). Enzymatic telomerase activity determined by TRAP assay revealed significantly higher activity in *TERT* rearranged and MNA cases (n = 9) as compared to other cases (n = 6; mean relative telomerase activity, 720 versus 164, p = 0.002, Additional file 4: Fig. S1C).

In line with our previous studies, we defined neuroblastomas as telomerase positive if *TERT* expression was above the threshold, if they harbored a *TERT* RA, or if they were *MYCN* amplified, as the latter had been shown to directly upregulate *TERT* expression in neuroblastoma [10, 13].

### Assessment of alternative lengthening of telomeres

Activation of ALT was assessed by detection of APB and by CCA. APB were observed in 27.9% of the tumors (19/68; Fig. 1B). As CCA results depend on a threshold of a reference sample, we investigated the impact of various published experimental strategies on the detection of ALT (Additional file 5: Fig. S2A and B) [22, 23]. According to this approach, we defined two distinct thresholds and determined the overlap of CCA with APB results (Additional file 5: Fig. S2B). The first threshold (th_1_) considered samples as ALT-positive in which C-circle signal intensity was ≥ 5% relative to the signal of the ALT-positive neuroblastoma cell line CHLA-90 [9]. The second threshold (th_2_) defined the level for CCA positivity as ≥ 20% of signal intensity of CHLA-90 and at least fourfold the area under the curve of polymerase-free dot-blot [12]. We found that the two thresholds revealed 16 and 12 ALT-positive cases respectively. Th_1_ revealed a larger overlap with results from APB analysis (94.0%, 63/67; Additional file 5: Fig. S2B), and was therefore selected for further analysis (Additional file 5: Fig. S2C).

### Classification of neuroblastoma samples according to TMM

Based on these results, we classified all neuroblastoma samples into three subgroups according to their TMM status (Fig. 1C): (i) Telomerase-positive tumors (TEL^**[+]**^), defined by the presence of *MNA*, *TERT* RA, or high *TERT* expression, and absence of APB or C-circle positivity; (ii) ALT-positive tumors (ALT^**[+]**^), defined by the absence of alterations associated with telomerase activation and by the presence of concordant positive results in APB analysis and CCA; (iii) TMM-negative tumors (TMM^**[−]**^) defined by the absence of all these alterations. According to these definitions, 89.7% of the tumors (61/68) were classified unambiguously (TEL^**[+]**^**,** n = 31; ALT^**[+]**^**,** n = 13; TMM^**[−]**^**,** n = 17; Fig. 1B and C). Of the remaining seven cases, two had high *TERT* expression and characteristics of ALT, and four had discordant results for APB and CCA. One tumor could not be classified unambiguously as only results for APB but not for CCA were available due to material shortage.

### Characteristics of TMM subgroups

To identify criteria that may help to unambiguously classify tumors into TEL^**[+]**^, ALT^**[+]**^, and TMM^**[−]**^ subgroups, we determined a panel of additional features that have been associated with telomere maintenance mechanisms previously (Fig. 1C and Additional file 6: Fig. S3).

### Mutations associated with the ALT phenotype

We found *ATRX* mutations in 8/68 samples (11.8%), 6 of which were classified as ALT^**[+]**^ neuroblastomas, and 2 of which were ambiguous. In ALT^**[+]**^ tumors, we observed deletions affecting exons 2–9 in two of the tumors, and deletions of exons 2–10 in two other tumors, one of which showed an additional deletion of exons 11–12. Furthermore, a nonsense mutation at position (c.853G > T) was detected. The largest deletion affected exons 2–15. In the two *ATRX* mutated tumors with ambiguous TMM status, we detected a deletion of exons 2–10 in one of these, and a deletion of exons 2–9 in the other one. We did not observe any mutations in the *ATRX* complex partner genes *DAXX* and *H3F3A* [24] nor mutations in *SMARCAL1* [25, 26].

### Long telomeres and a high telomere content are associated with ALT^[+]^ in neuroblastoma

**Fig. 2:**
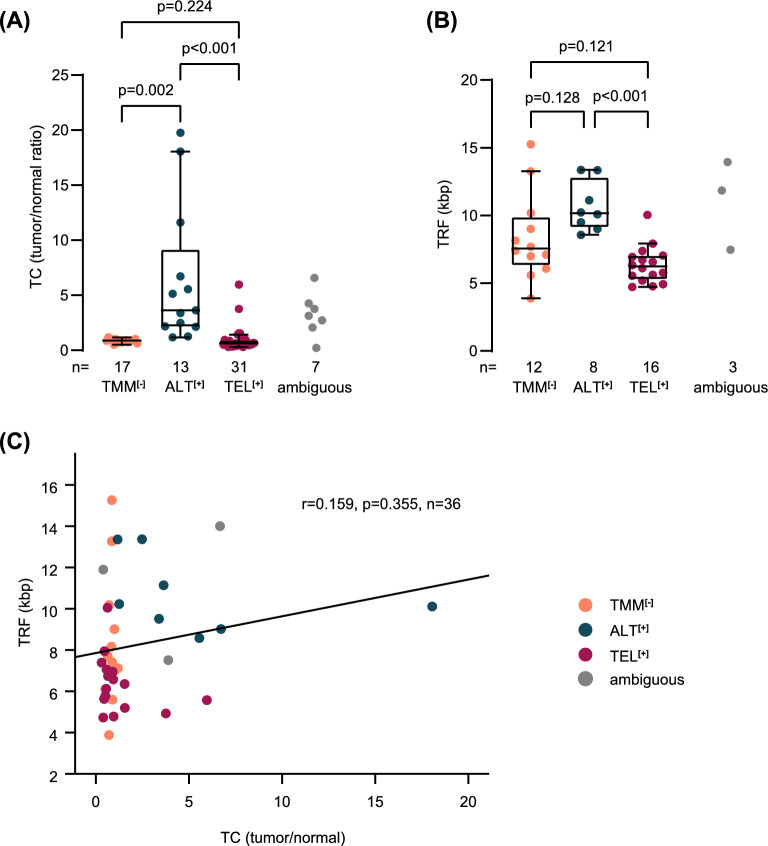
Telomere content and telomere restriction fragment analysis in TMM subgroups. **A** Telomere content (TC) calculated from WGS data, displayed as tumor/normal ratio, in telomere maintenance defined subgroups. Mean telomere content: TMM^**[−]**^, 0.87; ALT^**[+]**^, 6.39; TEL^**[+]**^, 0.96. Kruskal–Wallis test and Dunn’s multiple comparison test were used for statistical analysis; ambiguous cases were excluded. Whiskers are limited to 1.5 × interquartile range. **B** Telomere restriction fragment (TRF) analysis, displayed in kilobase pairs (kbp), in telomere maintenance defined subgroups. Mean telomere length: TMM^**[−]**^, 8.39; ALT^**[+]**^, 10.66; TEL^**[+]**^, 6.36. Kruskal–Wallis test and Dunn’s multiple comparison test were used for statistical analysis; ambiguous cases were excluded. Whiskers are limited to 1.5 × interquartile range. **C** Correlation analysis of TRF and TC in neuroblastoma samples (n = 36). Ambiguous cases were excluded from calculation of Pearson correlation

Since abundant telomeric sequences are characteristic for the ALT phenotype [27], we calculated the telomere content by inferring telomeric reads from WGS data (n = 68, Additional file 2: Tab. S2). Telomere content can reliably be calculated over a broad range of sequencing coverage and is positively correlated (linearly in unaltered normal controls) with read depth (Additional file 7: Fig. S4A). The telomere content, which was determined as the ratio between tumor and normal counts, was significantly higher in ALT^**[+]**^ (n = 13) than in TEL^**[+]**^ (n = 31) or TMM^**[−]**^ neuroblastomas (n = 17, Fig. 2A). Considering that matched normal samples might not always be available in clinical practice, we examined whether the telomere content computed from tumor samples only would provide similar results. We found that the telomere content of tumors only was also strongly associated with the ALT phenotype and was highly correlated with telomere content ratios of tumor/normal pairs (Additional file 7: Fig. S4B and C). We also determined telomere restriction fragment lengths by southern blot analysis (n = 39). Similarly to telomere content, telomere restriction fragments of ALT^**[+]**^ neuroblastomas (n = 8) were significantly longer than those of TEL^**[+]**^ tumors (n = 16), whereas they did not differ significantly between ALT^**[+]**^ and TMM^**[−]**^ cases (n = 12, Fig. 2B). Although telomeres were longest in ALT^**[+]**^ tumors in both analyses, the results of the two methods did not correlate significantly (Fig. 2C), suggesting that these assays may be complementary in detecting ALT-positive cases. In addition to the longer mean telomere restriction fragments, ALT^**[+]**^ tumors appeared to have more heterogeneous telomere restriction fragments (Additional file 8: Fig. S5).

**Fig. 3:**
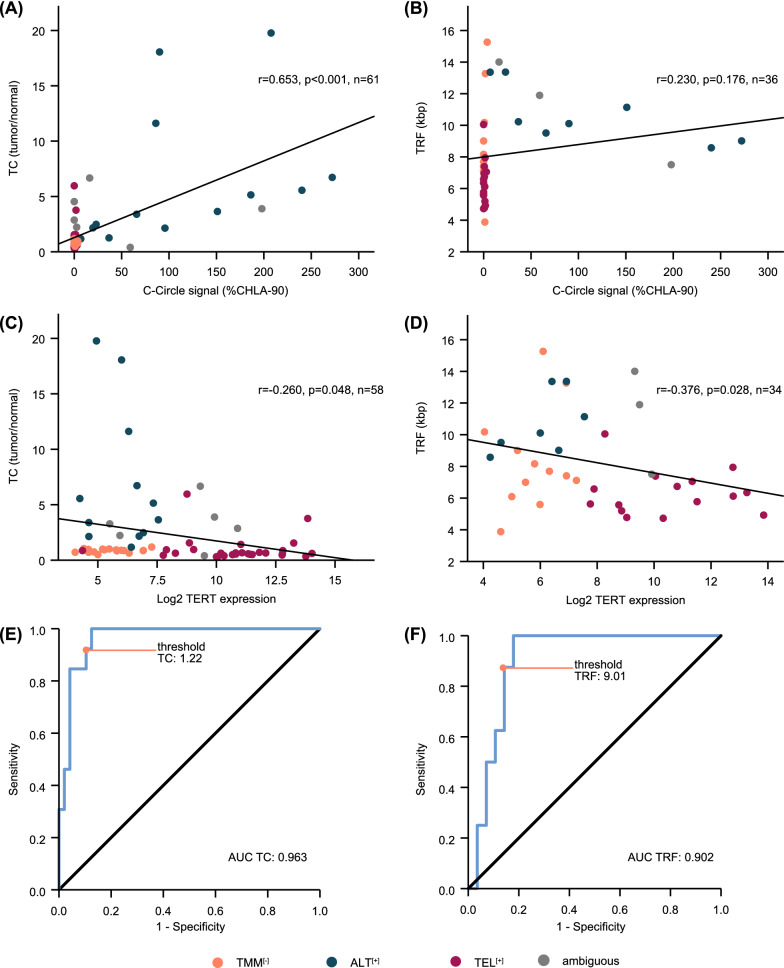
Association of telomere content and telomere restriction fragment lengths with ALT associated variables and ALT status. **A** Correlation analysis of telomere content (TC), displayed as tumor/normal ratio, and C-circle signal intensity. **B** Correlation analysis of telomere restriction fragment (TRF) lengths, displayed in kilobase pairs (kbp), and C-circle signal intensity. **C** Correlation analysis of TC and log_2_ *TERT* mRNA expression levels. **D** Correlation analysis of TRF and log_2_ *TERT* mRNA expression levels. Correlation coefficients were calculated according to Pearson; ambiguous cases were excluded from calculations. **E** Receiver operating curve (ROC) for ALT prediction by TC in neuroblastoma samples (n = 61). **F** ROC for ALT prediction by TRF in neuroblastoma samples (n = 36)

We further examined whether telomere content and telomere length correlated with other ALT-associated parameters. C-Circle expression intensity was positively correlated with telomere content (p < 0.001, Fig. 3A), but not with the lengths of telomere restriction fragments (p = 0.176, Fig. 3B). By contrast, both telomere content and telomere restriction fragments were negatively correlated with *TERT* expression (p = 0.048 and p = 0.028, Fig. 3C, D).

We also investigated the performance of telomere content and telomere length in identifying ALT in neuroblastoma. To this end, we computed receiver operating characteristics (ROC) curves on the cohort of cases with unambiguous TMM status and calculated the area under the curve (Fig. 3E, F). The AUC was ≥ 0.9 for both methods, suggesting that both are well suited to discriminate between ALT-positive and -negative cases [28]. To assess whether a combination of the two methods might further improve ALT detection, we performed binary logistic regression and calculated predicted probabilities for the combined ROC curve and AUC. A combination of telomere content and telomere length, however, did not improve ALT detection over telomere content alone substantially (Additional file 9: Fig. S6A). Based on the calculated coordinates of ROC curves, we propose a threshold of 1.22 (sensitivity 92.3%, specificity 89.6%, Fig. 3E) for detection of ALT by telomere content, and a threshold of 9.01 kb (sensitivity 87.5%, specificity 85.7%, Fig. 3F) for detection of ALT by telomere length in independent neuroblastoma cohorts (Additional file 3: Tab. S3).

### TERRA expression is elevated in ALT-positive neuroblastoma

**Fig. 4:**
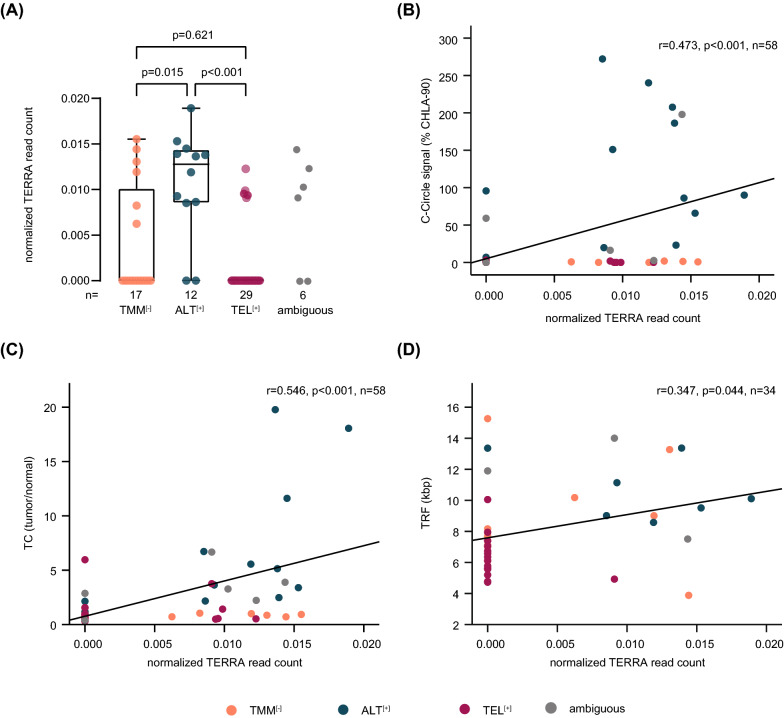
Association of TERRA expression and other ALT associated characteristics. **A** Normalized TERRA read count in telomere maintenance defined subgroups. Mean TERRA read count: TMM^**[−]**^, 0.004; ALT^**[+]**^, 0.011; TEL^**[+]**^, 0.002. Kruskal–Wallis test and Dunn’s multiple comparison test were used for statistical analysis; ambiguous cases were excluded. Whiskers are limited to 1.5 × interquartile range. **B** Correlation analysis of normalized TERRA read count and C-circle signal intensity. **C** Correlation analysis of TC, displayed as tumor/normal ratio, and normalized TERRA read count. **D** Correlation analysis of TRF, displayed in kilobase pairs (kbp), and normalized TERRA read count. Correlation coefficients were calculated according to Pearson; ambiguous cases were excluded from calculations

The expression of telomeric long non-coding RNA (TERRA) is associated with ALT in childhood neuroblastoma [12]. We, therefore, determined normalized TERRA read counts in TMM subgroups and found that TERRA was significantly higher in ALT^**[+]**^ compared to TMM^**[−]**^ and TEL^**[+]**^ neuroblastomas (Fig. 4A, Additional file 2: Tab. S2). Furthermore, TERRA expression correlated significantly with the intensity of the C-circle signal (p < 0.001, Fig. 4B), with telomere content (p < 0.001, Fig. 4C) and with telomere restriction fragment lengths (p = 0.044, Fig. 4D). To assess the performance of TERRA expression in predicting ALT, we computed the ROC curve also for this variable. The AUC was 0.826 and thus substantially inferior to both the AUC of ROC curves determined for telomere content or telomere restriction fragment lengths (Additional file 9: Fig. S6B).

### Fraction of telomere variant repeats (TVR) singletons are depleted in ALT-positive neuroblastoma

**Fig. 5:**
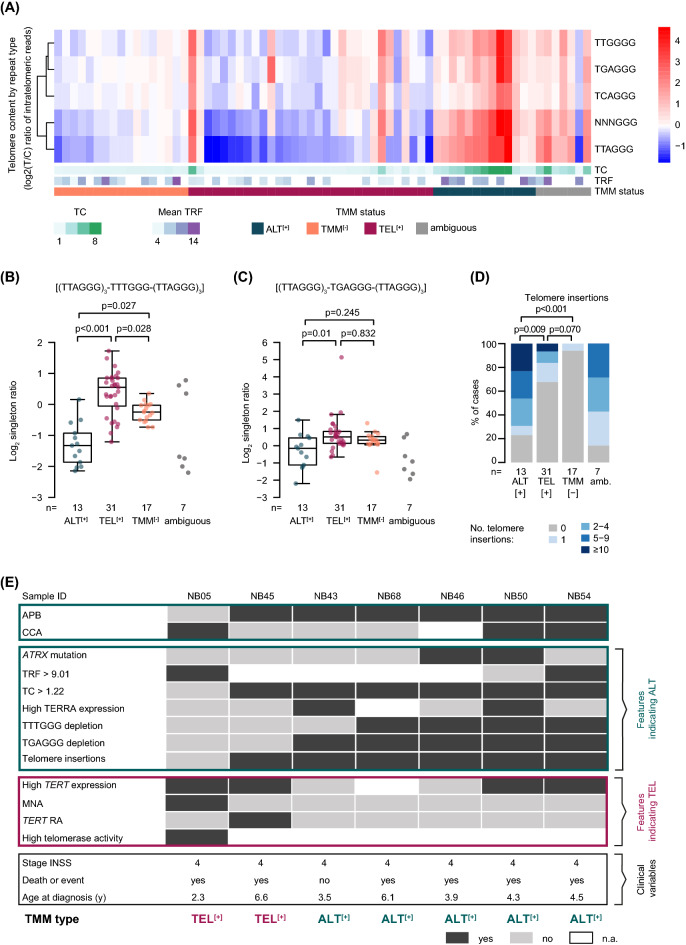
Additional features for TMM classification of ambiguous neuroblastoma cases. **A** Heatmap showing the telomere content of distinct telomere variant repeat types in neuroblastoma samples (n = 68). **B** Log_2_ singleton ratio for TTTGGG and **C** TGAGGG singletons, normalized by telomere content, in TMM defined subgroups. Kruskal–Wallis test and Dunn’s multiple comparison test were used for statistical analysis; ambiguous cases were excluded. Whiskers are limited to 1.5 × interquartile range. **D** Telomere insertions in TMM defined subgroups. Kruskal–Wallis test and Dunn’s multiple comparison test were used for statistical analysis; ambiguous cases were excluded from statistical analysis. No., number; amb., ambiguous cases. **E** TMM classification of seven ambiguous neuroblastoma cases that could not be classified by standard criteria. High TERRA expression was defined as equal or greater than the mean TERRA expression in ALT-positive neuroblastomas. TVR singleton depletion was defined as equal or smaller than the mean singleton ratio in ALT-positive neuroblastomas. Tumors were classified depending on the given TMM associated features into TEL^**[+]**^ or ALT^**[+]**^ subgroups. The clinical course was not considered for classification and is displayed for information only

Telomeres of cancer cells of distinct TMM subtypes may differ in their composition and content of so-called telomere variant repeats (TVR), which are variations of the most common telomeric hexamer (TTAGGG, t-type) [29]. We, therefore, examined telomeric reads for the most common (TTAGGG, TGAGGG, TCAGGG, TTGGGG) and all other variants (NNNGGG) of TVRs (Additional file 2: Tab. S2). Overall, all variants were more prevalent in ALT^**[+]**^ neuroblastoma (Fig. 5A). Another study has reported that TVR ‘singletons’, which are defined as a telomeric hexamer of the NNNGGG type surrounded by at least three t-type repeats on either side [(TTAGGG)_3_ – NNNGGG – (TTAGGG)_3_], may be more suitable to discriminate between TMM subgroups [8]. It has to be considered, though, that singleton counts increase with higher overall telomere content [8]. We thus screened telomeric reads for TVR singletons and compared the fraction of singleton counts in distinct TMM subtypes after normalization to telomere content. We observed a strong depletion of the TTTGGG singleton in ALT^**[+]**^ neuroblastoma (n = 13, p < 0.001, Fig. 5B), which is in line with a previous pan-cancer study [8]. We also found that the TGAGGG singleton was depleted in ALT^**[+]**^ neuroblastomas (n = 13) compared to TEL^**[+]**^ cases (n = 31, p = 0.013, Fig. 5C), whereas it was enriched in ALT-positive samples across multiple cancer types [8].

### Telomere insertions occur at high frequencies in ALT-positive neuroblastoma

It has been shown recently that intrachromosomal insertions of telomeric DNA correlate with ALT-associated mutations across multiple cancer types [8]. We, therefore, used WGS data to systematically screen for telomere insertions and examined potential associations with the distinct TMM-subgroups (Additional file 2: Tab. S2). Overall, we detected 134 breakpoints, representing 133 telomere insertions that affected 40% of the samples (27/68). The vast majority of insertions were defined as one-sided (99%, 132/133), meaning that only one breakpoint per insertion was detected, while two breakpoints in opposite directions were detected for only one insertion. The number of telomere insertions significantly correlated with telomere content (spearman correlation, r = 0.407, p < 0.001; Additional file 10: Fig. S7A). The prevalence of telomere insertions differed considerably between TMM-subgroups: 77% (10/13) of ALT^**[+]**^ samples harbored telomere insertions, whereas the fraction was 32% (10/31) and 6% (1/17) in TEL^**[+]**^ and TMM^**[−]**^ samples, respectively (Fig. 5D). We also noted that all samples bearing *ATRX* mutations harbored at least one telomere insertion and thus had the highest frequency of such alterations (Additional file 10: Fig. S7B).

### Classification of ambiguous cases into TMM subgroups

We finally aimed to determine the TMM status in those seven cases that had remained ambiguous in our initial classification. In detail, 4/7 cases had discordant results in CCA and APB analysis, whereas only APB but no CCA was available in one case (Additional file 11: Fig. S8). Two cases of these showed high *TERT* expression despite the presence of either APB or C-circles, and two additional cases had high *TERT* expression despite the presence of both APB and C-circles. To define the TMM status of these cases, we took all available information into account, i.e., genomic alterations, *TERT* expression, telomerase activity, CCA and APB results, telomere content and telomere length, TERRA expression, TVR singletons and telomere insertions (Fig. 5E). In case NB05, which was CCA-positive and APB-negative, we detected MNA, high *TERT* expression and high telomerase activity, whereas the telomere content and TERRA expression were low and no ALT-associated TVR depletion or telomere insertions were found, suggesting that NB05 was TEL^**[+]**^. Case NB45 was CCA-negative and ABP-positive, and showed a high telomere content and telomere insertions while lacking TERRA expression. However, as this tumor harbored a *TERT* rearrangement and high *TERT* expression, we considered this case as TEL^**[+]**^. In case NB43, which also was CCA-negative and ABP-positive, we detected TERRA expression, a high telomere content, telomere insertions and low *TERT* expression, supporting the notion that this case was ALT^**[+]**^. Similarly, case NB68 was CCA-negative and APB-positive and had a particularly high telomere content, telomere insertions, and a low fraction of TVR singletons along with no evidence of *TERT* activation, suggesting that this tumor was ALT^**[+]**^. NB46, for which only APB was available, showed an *ATRX* mutation, high telomere content, TVR singleton depletion and telomere insertions, and was thus likely to be ALT^**[+]**^. In NB50, we detected high *TERT* expression, however, many other features, i.e., CCA, APB, *ATRX* mutation, telomere content, TERRA expression, telomere insertions, and a low fraction of TVR singletons supported an ALT^**[+]**^ phenotype. Similarly, we found multiple features of ALT in NB54 (CCA, APB, high telomere content, long telomere restriction fragments, low fraction of TVR singletons and one telomere insertion) despite high *TERT* expression, thus pointing towards an activated ALT^**[+]**^ pathway. Together, two of the ambiguous cases were classified as TEL^**[+]**^ and five as ALT^**[+]**^.

### Workflow for TMM assessment in neuroblastoma

**Fig. 6:**
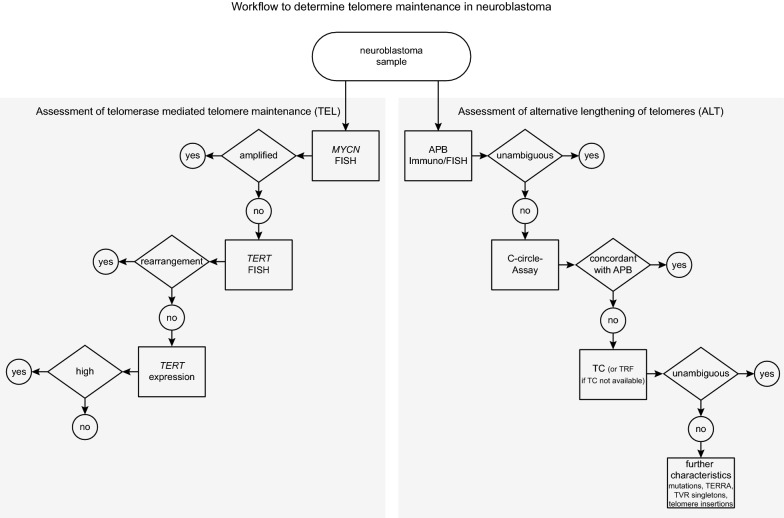
Workflow for assessment of telomere maintenance in neuroblastoma

Taken together, our data indicate that assessment of multiple parameters related to TMM allows determining the TMM status also in those neuroblastoma cases that have remained ambiguous. In our cohort, CCA was false negative in two cases (NB43 and NB68) and false positive in one case (NB05), whereas APB analysis was false positive in one case (NB45, Fig. 5E). Based on these findings, we propose a workflow to reliably determine TMM in neuroblastoma, taking into account a stepwise approach of diagnostic assays (Fig. 6).

## Discussion

Maintenance of telomeres is a hallmark of high-risk neuroblastoma [6, 10]. Activation of TMM is mediated via a telomerase-dependent or via an alternative, telomerase-independent pathway [6]. Despite its potential prognostic value, assessment of TMM is not part of the clinical routine in neuroblastoma patients yet [30, 31], and no guidelines have been established on how TMM should be determined. Here, we propose a stepwise diagnostic workflow for TMM assessment in neuroblastoma based on extensive characterization of primary tumor samples. The workflow also considers individual conditions of clinical centers, like availability of material, access to NGS techniques, and experience of investigators (Fig. 6).

For assessment of telomerase activation, we recommend determining *MYCN* copy number status by FISH as the first step, which is a well-established method and part of the clinical routine for decades [30]. In *MYCN* non-amplified tumors, we recommend subsequently determining the genomic status of *TERT* by FISH or by WGS, if available [10, 32]. This stepwise approach will identify the vast majority of TEL^**[+]**^ tumors. If neither MNA nor *TERT* RA are detected, we recommend determining *TERT* expression levels by RNA sequencing or RNA expression microarrays to identify cases with elevated *TERT* expression but lacking corresponding genomic alterations.

Detection of ALT is more challenging, as the underlying mechanisms have remained largely elusive [33], and ALT-positivity is thus identified by analysis of phenotypic characteristics that are associated with ALT in the majority, but not necessarily all cases (e.g., C-circles [9, 17], APB [9, 16] or *ATRX* mutations [12, 18]). Detection of C-circles or APB is frequently used for ALT assessment [6, 9, 12], however, no consensus exists on ALT detection in neuroblastoma. We evaluated both methods and found that the two assays revealed identical results in all except four cases (94%, 63/67). Discordant results of APB detection and CCA have been reported also in previous studies [9, 16, 17, 34, 35]. Although C-circle detection is supposed to be highly specific for ALT-positive cells, low signals can be obtained also from normal healthy tissue [36], especially from blood samples [22] (Additional file 5: Fig. S2A). Likewise, occasional formation of APB in ALT-negative cells, such as small APB-like bodies in healthy tumor-adjacent tissue, has been reported [37, 38]. Thus, both methods lack specificity and sensitivity in detecting ALT. In our study, APB detection appeared to perform slightly more accurately than CCA, since only one sample was misclassified by APB analysis, whereas three were misclassified by CCA. Another potential advantage of APB analysis may be the fact that this method is based on microscopic evaluation of tumor tissue sections, which may allow the identification of intratumoral heterogeneity. On the other hand, APB detection can be challenging in some instances, and thus requires a certain extent of investigator experience. A potential limitation of CCA may be the inconsistency in definitions of appropriate detection thresholds and reference cell lines [9, 12, 16, 17, 35, 39], as different thresholds may lead to different results (Additional file 5: Fig. S2B). By contrast, the continuous nature of C-circle signals may be advantageous for quantitatively monitoring ALT activity in longitudinal samples [34, 40, 41]. Taken together, we consider both APB detection and CCA as appropriate for ALT assessment as a first step. Because of the considerations mentioned above, however, we prioritize APB analysis if appropriate expertise is available, and recommend performing CCA additionally, at least in cases with inconclusive APB results.

If the ALT status cannot be determined unequivocally by APB analysis and CCA, we recommend analyzing telomere lengths as a next step, preferentially by telomere content assessment using WGS data. In our study, telomere content and—to a lesser extent—telomere restriction fragments correlated well with ALT-associated markers, such as C-circle and low *TERT* expression, supporting the association of ALT and long telomeres [8, 11, 12, 27]. Both methods may have limitations: Telomere restriction fragment analysis can be affected by the variability of sub-telomeric regions and hybridization issues, whereas calculation of the telomere content can be imprecise due to unstable karyotypes of cancer cells [42]. We found, however, that both methods may be suitable for ALT assessment in neuroblastoma if appropriate thresholds are being used. We also showed that telomere content parameters can be inferred from relatively low sequencing coverage [43] and from tumor WGS data only, without the necessity to sequence matched normal DNA.

In difficult-to-classify cases, we recommend considering additional analyses to further evaluate ALT-associated characteristics. Expression of TERRA has been associated with ALT-positive cancers [8, 12, 44], which was also observed in this study. We further show that examination of telomeric variant repeats [8] may be helpful for determine the TMM status, as they differ in content and composition dependent on ALT status [29, 45, 46]. In addition, we found enrichment of telomere insertions in ALT^**[+]**^ cases in our study, which have previously been shown to accumulate in cancers with ALT-associated mutations and to correlate with telomere content [8]. Finally, detection of ALT-associated mutations may contribute to determine the ALT status in neuroblastoma, as reported for other cancer types [8, 44]. Such analyses, however, can merely supplement other diagnostic assays, as they may have a high specificity, but low sensitivity for detection of ALT [12, 18]. Thus, detection of ALT-associated genomic mutations may be complementarily used to confirm ALT positivity, however, the absence of such mutations does not substantiate ALT negativity.

## Conclusions

We here present a diagnostic workflow to reliably assess TMM in primary neuroblastoma samples. Our study thus may guide neuroblastoma reference laboratories in setting up appropriate diagnostic assays, provide a framework for establishing comparability of TMM results, and ultimately guide pediatric oncologists in accurate risk assessment of neuroblastoma patients.

## Methods

### Patients and cohort

We retrospectively analyzed primary neuroblastoma samples, for all of which whole genome sequencing (WGS) data were available. Samples were obtained from patients enrolled in the clinical trials NB97 (n = 14), NB2004 (n = 51) or NB2016 Registry (n = 3) of the Gesellschaft für Pädiatrische Onkologie und Hämatologie (GPOH). Informed consent was given from all patients or their guardians. The Institutional Review Board of the Medical Faculty of the University of Cologne granted the ethical approval for the use of specimens. Since the availability of WGS data was our defining criteria for the cohort, part of the information on patient samples had been published in previous studies [10, 21, 47]. Most patients were diagnosed with Stage 4 disease according to INSS (66.2%, n = 45), however, all other stages were represented in the cohort (Stage 1: 11.8%, Stage 2: 10.3%, Stage 3: 4.4%, Stage 4S: 7.4%, Additional file 1: Tab. S1). The gender ratio was distributed with 54.4% male and 45.6% female patients.

To detect and assess TMM in neuroblastoma, we applied different experimental and sequencing-based approaches. *MYCN* copy number was obtained from a routine diagnostic workup by FISH (n = 68). *TERT* RA were called from WGS data (n = 68), partly supplemented by FISH to visualize the rearrangements (n = 23). *TERT* expression was obtained from RNA sequencing data (n = 64). The threshold for defining high versus low *TERT* expression was calculated on RNA sequencing data of a large neuroblastoma cohort [21] as described previously for microarray analysis [6]. Samples were considered ‘high’ if *TERT* expression was above 7.58 (Additional file 4: Fig. S1A). ALT detection was based on APB Immuno/FISH (n = 68) and CCA (n = 67). Telomere lengths were obtained from telomere restriction fragment (TRF) analysis (n = 39), or telomere content (TC) was calculated from WGS data (n = 68). TERRA expression was calculated from RNA sequencing data (n = 64). Telomere insertions, telomere variant repeats (TVR) content and composition, and ALT-associated somatic mutations were called from WGS data (n = 68). Telomerase activity was determined by TRAP assay (n = 15).

### WGS data analyses

Paired-end WGS data was available for the whole cohort and part of WGS data analysis was published previously [10, 47]. Library preparation and data analysis (i.e., sequence alignment, mutation and rearrangement calling) were carried out as reported before [10]. Mean read depth was between 24 and 65 × for tumor and 11 and 49 × for normal samples (Additional file 7: Fig. S4A, Additional file 2: Tab. S2). Telomere content was estimated by counting reads containing at least four times the most common t-type repeat sequence (TTAGGG or its reverse complement) in paired tumor and normal samples. The counts were further normalized by the total number of reads in the sample and the final estimates for each patient are given as the ratio between tumor and normal. WGS with read depths around 10x (Additional file 7: Fig. S4A) or, alternatively, WES, is sufficient to obtain sufficient reads to calculate telomere content statistics [10, 43]. If no (matched) normal samples are available, the normalized telomeric read counts of single tumor samples are still sufficient to distinguish ALT positive cases comparable to matched tumor/normal pairs (Additional file 7: Fig. S4B and C). Similar ratios of other TVR sequences of type NNNGGG and TVR-singletons were computed with TelomereHunter [48] (Version 1.0.4) using default parameters. Singletons were defined as TVRs flanked by three t-type repeats [(TTAGGG)_3_-NNNGGG-(TTAGGG)_3_] and their ratios were further divided by telomere content for comparison between TMM subgroups.

For telomeric insertion detection, an approach by Siverling et al. [8] was adapted. In detail, the TelomereHunter output was used to identify ‘telomere insertion’ read pairs, where one mate was classified as telomere read and the other mate mapped onto the genome in a non-telomeric or -centromeric region. Next, the genome was split up into 1 kb windows and windows with at least three previously identified ‘telomere insertion’ read pairs in the tumor sample and none in the normal were considered as approximate candidates for insertion sites. Every candidate window was then searched for soft-clipped alignments marking the exact breakpoint. These ‘telomeric’ soft-clipped sequences were required to be at least 15 bp long, contain at least two t-type repeats (TTAGGG or the reverse complement) and start at the same position (± 1 bp). Breakpoints with at least two ‘telomeric’ soft-clipped reads were then considered for a final visual inspection using the IGV [49, 50] to remove false positive detections (e.g., in regions with simple nucleotide repeats or t-type repeats in the reference genome). While it is possible to omit the comparison to a matched normal sample, it is advised to use matched tumor/normal pairs to reduce the number of false-positive candidates.

### RNA sequencing and TERRA expression analysis

Part of the RNA sequencing data had been published previously [21]. Gene expression was calculated as previously reported, using AceView Magic pipeline [10, 21] and AceView transcriptome reference (http://www.aceview.org). TERRA expression was computed by first mapping the paired-end reads to the hg19 reference with STAR [51], and using TelomereHunter [48] with default parameters to count reads containing the four most common TVRs (TTAGGG, TGAGGG, TCAGGG, TTGGGG). Individual counts were further normalized by the total number of reads and multiplied by 10^6^. The total normalized TERRA expression in a sample was defined as the sum of the four individual TERRA values.

### TERT break-apart FISH

*TERT* rearrangements were detected as published previously [10]. Customized digoxigenin and biotin-labeled FISH probes were used for hybridization. Streptavidin-Alexa-555 conjugate (1:500 in CAS-block, Invitrogen, S21381), and anti-digoxigenin-FITC (1:500 in CAS-block, Roche, 11 207 741 910) antibodies were used. Slides were counterstained with DAPI (containing 4′,6-diamidino-2-phenylindole dihydrochloride, Vectorlab, H-1200–10). Microscopy was performed using a Leica DM5500 system with Cytovision (Leica, version 7.7) and FIJI (version 1.52p) software tools.

### Combined immunofluorescence and FISH for APB

APB were detected by combined immunofluorescence and FISH as published previously with slight changes [52]. Slides were washed and fixed with 2% Paraformaldehyde. Graded ethanol dehydration was followed by the application of a telomere PNA probe (Tel C-Alexa-Fluor-488, PNA). Denaturation was done at 75 °C and hybridization was induced at 37 °C overnight. After washing, permeabilization and blocking, the primary antibody for immunofluorescence was applied (1:250 in blocking solution, PML antibody, rabbit, H-238, Santa Cruz Biotechnology, sc-621) at 4 °C overnight. The incubation with secondary antibody (1:2500 in 1X PBS, goat anti-rabbit Alexa Fluor 555, Invitrogen by Thermo Fisher Scientific, A27039), was followed by counterstaining with DAPI. Microscopy was performed using a Leica DM5500 system with Cytovision (Leica, version 7.7) and ImageJ, FIJI (version 1.52p) software tools.

### C-circle assay

The CCA was performed according to previously published protocols with few changes [36]. 60 ng of genomic DNA were digested at 37 °C for 1 h with restriction enzymes Hinf I, RSA l and RNAse, such that telomeric DNA including the C-circle molecules remained intact. Rolling circle amplification by Φ 29 polymerase (New England Biolabs, M0269S) was performed at 30 °C. The amplification products and a negative control for each sample without polymerase were dot-blotted. Further steps including hybridization to a DIG-labelled telomere probe and detection of the chemiluminescent signal were performed using the Telo-TAGGG Telomere Length Assay kit (Roche, 12 209 136 001) according to the manufacturer’s protocol.

### Telomere length assay

The mean TRF was determined by the Telo-TAGGG Telomere Length Assay kit (Roche, 12 209 136 001) according to the manufacturer’s instructions. The optical density (OD) was obtained for each position *i* with the image processing software FIJI (Version 1.52p). Mean TRF was calculated by the equation: mean TRF = ∑ (OD *i* * L*i*) / ∑ (OD *i*) where L*i* is the length of the TRF at position *i* [53].

### Telomerase activity

Telomerase activity was assessed as published previously [6] by the TeloTAGGG Telomerase PCR ELISA^PLUS^ Kit (Sigma Aldrich, 12,013,789,001) according to the manufacturer`s instructions.

### Cell lines

SK-N-FI cells were obtained from ATCC (Manassas, VA, USA), and CHLA-90 cells were obtained from the Children's Oncology Group (COG) Cell Culture and Xenograft Repository. Lan-6 and NBL-S were purchased from DSMZ (Braunschweig, Germany). KELLY and SK-N-BE(2) cells were kindly provided by Dr. Olaf Witt and CLB-GA by Dr. Johannes Schulte. Cell lines were validated by the DSMZ using STR profiling. LM-216-J were kindly provided by Dr. Roderick O`Sullivan. CHLA-90 cells were cultured in IMDM (Thermo Fisher, 12,440–053) supplemented with 20% heat-inactivated fetal bovine serum (Gibco, 10,500–064) and 1% Insulin–transferrin–sodium selenite (ITS) media supplement (Sigma Aldrich, I1884). NBL-S were cultured in IMDM with 10% heat-inactivated fetal bovine serum. SK-N-FI were cultured in DMEM (Thermo Fisher, 11,995–065), 10% heat-inactivated fetal bovine serum and 1% non-essential amino acids (Thermo Fisher, 11,140,050). For Lan-6, DMEM was used supplemented with 20% heat-inactivated fetal bovine serum. Kelly, CLB-GA and SK-N-BE(2) cells were cultured in RPMI (Thermo Fisher, 61,870-010) supplemented with 10% heat-inactivated fetal bovine serum. Cells were grown at 37 °C in a humidified atmosphere with 5% CO_2_.

### Statistics

For statistical analysis and illustration of data IBM SPSS statistics (Version 27.0.0), R (Version 4.1.1), GraphPad Prism (Version 9.0.1) and Adobe Illustrator (Version 25.2) were used. Pearson test was used to detect correlations between metrically scaled variables, e.g., telomere content, mean TRF, TERRA read count, C-circle signal, and *TERT* expression levels. Spearman correlation was used to detect correlation between telomere content and the number of telomere insertions. Comparisons between groups were computed with the Kruskal-Wallis test followed by Dunn’s multiple comparison test. Differences between means of metric, normally distributed variables were tested by unpaired t-test or by ANOVA followed by Tukey post-hoc test if more than two subgroups were analyzed.

## Supplementary Information

**Additional file 1: Table S1.** Neuroblastoma patient characteristics.

**Additional file 2: Table S2.** Sequencing based metrics of neuroblastoma samples.

**Additional file 3: Table S3.** Coordinates for ROC curve TC and TRF.

**Additional file 4: Figure S1**. *TERT* expression and telomerase activity in neuroblastoma with activated telomere maintenance mechanism. (A) Distribution of *TERT* log_2_ expression values, determined by RNA sequencing, in neuroblastomas harboring *TERT* rearrangements, and/or MNA or none of these alterations. The threshold at 7.58 was defined as the lowest expression value having a posterior probability ≥95% to fall within the distribution on the right (i.e., the group of tumors with *TERT*/*MYCN* alteration). (B) Log_2_ *TERT* mRNA expression levels, dependent on telomere maintenance subgroup. ANOVA, Tukey’s multiple comparison test. n=55, RNA sequencing data was available for 64 cases, however ambiguous cases (n=7) as well as cases that show MNA and *TERT* RA in the same tumor (n=3) were excluded (see also Fig. 1B). Whiskers are limited to 1.5x interquartile range. (C) Relative telomerase activity dependent on underlying alteration, determined by TRAP assay. Unpaired t-test. n=15. Whiskers are limited to 1.5x interquartile range.

**Additional file 5: Figure S2.** Dependency of C-circle assay on threshold and reference cell line. (A) Southern blot of C-circle assay of different cell lines (CHLA-90: ALT^[+]^, SK-N-BE(2): TEL^[+]^, SK-N-FI: ALT^[+]^, LM-216-J: ALT^[+]^), normal human tissue and neuroblastoma samples with high telomerase activity (all ALT-negative). Left columns: sample without polymerase, right columns: samples with polymerase. (B) Neuroblastoma samples for which APB and C-circle assay was available (n=67). Different thresholds applied to the same samples reveal different results. (th_1_) C-circle signal intensity ≥5% relative to the signal of CHLA-90. (th_2_) C-circle signal intensity ≥20% relative to the signal of CHLA-90 and at least fourfold the area under the curve of polymerase-free dot-blot. Number of cases (n) classified as ALT-positive according to the respective threshold are indicated. (C) Representative image of southern blot of C-Circle assay of different cell lines and neuroblastoma samples. Left columns: sample without polymerase, right columns: samples with polymerase. ALT-positive cell line CHLA-90 and ALT-negative cell line SK-N-BE are depicted at the top, ALT status according to threshold th_1_ as indicated.

**Additional file 6: Figure S3**. Venn diagram on availability of experimental data.

**Additional file 7: Figure S4.** Detection of telomeric reads and calculation of telomere content on WGS data. (A) Number of detected telomeric reads, i.e., reads containing at least four t-type repeats, in tumor and normal WGS data in relation to the mean read depth. Pearson correlation displayed for normal controls only. (B) Normalized telomere content calculated from single tumor WGS data, dependent on telomere maintenance subgroups. Mean telomere content: TMM^[-]^ 67.43, ALT^[+]^ 339.33, TEL^[+]^ 54.19. Kruskal-Wallis test and Dunn’s multiple comparison test, n=61, ambiguous cases were excluded from statistical analysis. Whiskers are limited to 1.5x interquartile range. (C) Correlation analysis of telomere content calculated from tumor/normal ratios and the respective single tumor samples.

**Additional file 8: Figure S5.** A distinct pattern of TRF southern blot analysis dependent on the TMM subgroup. Southern blot for telomere restriction fragment analyses of neuroblastoma samples and cell lines. ALT-positive cell line SK-N-FI and ALT-negative but telomerase-positive cell line Kelly were used on every blot with tumor samples as controls on every blot.

**Additional file 9: Figure S6. **Features for prediction of TMM status in neuroblastoma. (A) Combined ROC for TC and TRF. After binary logistic regression and calculation of predicted probabilities, used for combined ROC, n=36. (B) ROC for normalized TERRA read count as a classifier for ALT, n=58.

**Additional file 10: Figure S7. **Telomere insertions in ALT-positive neuroblastoma. (A) Correlation analysis of numbers of telomere insertions into non-telomere regions and telomere content calculated on basis of WGS data, n=68. Ambiguous cases were excluded for spearman correlation. (B) Telomere insertions in the entire cohort and in subgroups of tumors with *ATRX* mutations.

**Additional file 11: Figure S8.** Raw data of CCA and combined Immunofluorescence/FISH of ambiguous neuroblastoma cases. (A) Images of CCA and (B) combined Immunofluorescence/FISH of ambiguous neuroblastoma samples with contrasting results as revealed by the two methods. Images of Immunofluorescence/FISH show ultrabright telomeric signals (green) and associated APBs (red) in ALT-positive cases, whereas the ALT-negative sample (NB05) does not show prominent telomeric signals and only subtle PML bodies. (C) Example of one unambiguous ALT-negative and one unambiguous ALT-positive neuroblastoma.

## Data Availability

Patient whole-genome sequencing data are deposited at the European Genome-phenome Archive (https://ega-archive.org) under study accession numbers EGAS00001001308, EGAS00001005424 and EGAS00001006510. RNA sequencing data is deposited at GEO (https://www.ncbi.nlm.nih.gov/geo/) under accession number GSE49711 and GSE211653. Due to the sensitive nature of the patient datasets, the WGS data is subject to approval by the data provider. Please see the corresponding EGA data access committee (DAC) for more details on the procedure (https://ega-archive.org/dacs/EGAC00001000361).

## Abbreviations

ALT: Alternative lengthening of telomeres
APB: ALT-associated promyelocytic leukemia nuclear bodies
CCA: C-circle assay
FISH: Fluorescence in situ hybridization
GPOH: Gesellschaft für pädiatrische Onkologie und Hämatologie
IF: Immunofluorescence
MNA: MYCN amplification
RA: Rearrangement
TEL: Telomerase
TERRA: Telomeric long non-coding RNA
TMM: Telomere maintenance mechanisms
TRF: Telomere restriction fragment
TVR: Telomere variant repeats
WGS: Whole genome sequencing

## References

[1] Maris JM, Hogarty MD, Bagatell R, Cohn SL (2007). Neuroblastoma. Lancet.

[2] Matthay KK, Maris JM, Schleiermacher G, Nakagawara A, Mackall CL, Diller L, Weiss WA (2016). Neuroblastoma. Nat Rev Dis Primers.

[3] Simon T, Hero B, Schulte JH, Deubzer H, Hundsdoerfer P, von Schweinitz D, Fuchs J, Schmidt M, Prasad V, Krug B (2017). 2017 GPOH guidelines for diagnosis and treatment of patients with neuroblastic tumors. Klin Padiatr.

[4] Cohn SL, Pearson AD, London WB, Monclair T, Ambros PF, Brodeur GM, Faldum A, Hero B, Iehara T, Machin D (2009). The International neuroblastoma risk group (INRG) classification system: an INRG task force report. J Clin Oncol.

[5] Ora I, Eggert A (2011). Progress in treatment and risk stratification of neuroblastoma: impact on future clinical and basic research. Semin Cancer Biol.

[6] Ackermann S, Cartolano M, Hero B, Welte A, Kahlert Y, Roderwieser A, Bartenhagen C, Walter E, Gecht J, Kerschke L (2018). A mechanistic classification of clinical phenotypes in neuroblastoma. Science.

[7] Hanahan D, Weinberg RA (2011). Hallmarks of cancer: the next generation. Cell.

[8] Sieverling L, Hong C, Koser SD, Ginsbach P, Kleinheinz K, Hutter B, Braun DM, Cortes-Ciriano I, Xi R, Kabbe R (2020). Genomic footprints of activated telomere maintenance mechanisms in cancer. Nat Commun.

[9] Koneru B, Lopez G, Farooqi A, Conkrite KL, Nguyen TH, Macha SJ, Modi A, Rokita JL, Urias E, Hindle A (2020). Telomere maintenance mechanisms define clinical outcome in high-risk neuroblastoma. Cancer Res.

[10] Peifer M, Hertwig F, Roels F, Dreidax D, Gartlgruber M, Menon R, Kramer A, Roncaioli JL, Sand F, Heuckmann JM (2015). Telomerase activation by genomic rearrangements in high-risk neuroblastoma. Nature.

[11] Henson JD, Neumann AA, Yeager TR, Reddel RR (2002). Alternative lengthening of telomeres in mammalian cells. Oncogene.

[12] Hartlieb SA, Sieverling L, Nadler-Holly M, Ziehm M, Toprak UH, Herrmann C, Ishaque N, Okonechnikov K, Gartlgruber M, Park YG (2021). Alternative lengthening of telomeres in childhood neuroblastoma from genome to proteome. Nat Commun.

[13] Roderwieser A, Sand F, Walter E, Fischer J, Gecht J, Bartenhagen C, Ackermann S, Otte F, Welte A, Kahlert Y (2019). Telomerase is a prognostic marker of poor outcome and a therapeutic target in neuroblastoma. JCO Precis Oncol.

[14] Chen J, Nelson C, Wong M, Tee AE, Liu PY, La T, Fletcher JI, Kamili A, Mayoh C, Bartenhagen C (2021). Targeted therapy of TERT-rearranged neuroblastoma with BET bromodomain inhibitor and proteasome inhibitor combination therapy. Clin Cancer Res.

[15] Zhang JM, Zou L (2020). Alternative lengthening of telomeres: from molecular mechanisms to therapeutic outlooks. Cell Biosci.

[16] Henson JD, Cao Y, Huschtscha LI, Chang AC, Au AY, Pickett HA, Reddel RR (2009). DNA C-circles are specific and quantifiable markers of alternative-lengthening-of-telomeres activity. Nat Biotechnol.

[17] Plantinga MJ, Pascarelli KM, Merkel AS, Lazar AJ, von Mehren M, Lev D, Broccoli D (2013). Telomerase suppresses formation of ALT-associated single-stranded telomeric C-circles. Mol Cancer Res.

[18] Zeineldin M, Federico S, Chen X, Fan Y, Xu B, Stewart E, Zhou X, Jeon J, Griffiths L, Nguyen R (2020). MYCN amplification and ATRX mutations are incompatible in neuroblastoma. Nat Commun.

[19] Nersisyan L, Simonyan A, Binder H, Arakelyan A (2021). Telomere maintenance pathway activity analysis enables tissue- and gene-level inferences. Front Genet.

[20] Lu R, O'Rourke JJ, Sobinoff AP, Allen JAM, Nelson CB, Tomlinson CG, Lee M, Reddel RR, Deans AJ, Pickett HA (2019). The FANCM-BLM-TOP3A-RMI complex suppresses alternative lengthening of telomeres (ALT). Nat Commun.

[21] Zhang W, Yu Y, Hertwig F, Thierry-Mieg J, Zhang W, Thierry-Mieg D, Wang J, Furlanello C, Devanarayan V, Cheng J (2015). Comparison of RNA-seq and microarray-based models for clinical endpoint prediction. Genome Biol.

[22] Idilli AI, Segura-Bayona S, Lippert TP, Boulton SJ (2021). A C-circle assay for detection of alternative lengthening of telomere activity in FFPE tissue. STAR Protoc.

[23] Lau LM, Dagg RA, Henson JD, Au AY, Royds JA, Reddel RR (2013). Detection of alternative lengthening of telomeres by telomere quantitative PCR. Nucleic Acids Res.

[24] Schwartzentruber J, Korshunov A, Liu XY, Jones DT, Pfaff E, Jacob K, Sturm D, Fontebasso AM, Quang DA, Tonjes M (2012). Driver mutations in histone H3.3 and chromatin remodelling genes in paediatric glioblastoma. Nature.

[25] Diplas BH, He X, Brosnan-Cashman JA, Liu H, Chen LH, Wang Z, Moure CJ, Killela PJ, Loriaux DB, Lipp ES (2018). The genomic landscape of TERT promoter wildtype-IDH wildtype glioblastoma. Nat Commun.

[26] Cox KE, Marechal A, Flynn RL (2016). SMARCAL1 resolves replication stress at ALT telomeres. Cell Rep.

[27] Bryan TM, Englezou A, Gupta J, Bacchetti S, Reddel RR (1995). Telomere elongation in immortal human cells without detectable telomerase activity. EMBO J.

[28] Hosmer DWLS, Sturdivant RX (2013). Assessing the fit of the model applied logistic regression.

[29] Lee M, Hills M, Conomos D, Stutz MD, Dagg RA, Lau LM, Reddel RR, Pickett HA (2014). Telomere extension by telomerase and ALT generates variant repeats by mechanistically distinct processes. Nucleic Acids Res.

[30] Sokol E, Desai AV (2019). The evolution of risk classification for neuroblastoma. Children (Basel).

[31] Pinto NR, Applebaum MA, Volchenboum SL, Matthay KK, London WB, Ambros PF, Nakagawara A, Berthold F, Schleiermacher G, Park JR (2015). Advances in risk classification and treatment strategies for neuroblastoma. J Clin Oncol.

[32] Valentijn LJ, Koster J, Zwijnenburg DA, Hasselt NE, van Sluis P, Volckmann R, van Noesel MM, George RE, Tytgat GA, Molenaar JJ (2015). TERT rearrangements are frequent in neuroblastoma and identify aggressive tumors. Nat Genet.

[33] Cesare AJ, Reddel RR (2010). Alternative lengthening of telomeres: models, mechanisms and implications. Nat Rev Genet.

[34] Grandin N, Pereira B, Cohen C, Billard P, Dehais C, Carpentier C, Idbaih A, Bielle F, Ducray F, Figarella-Branger D (2019). The level of activity of the alternative lengthening of telomeres correlates with patient age in IDH-mutant ATRX-loss-of-expression anaplastic astrocytomas. Acta Neuropathol Commun.

[35] Dagg RA, Pickett HA, Neumann AA, Napier CE, Henson JD, Teber ET, Arthur JW, Reynolds CP, Murray J, Haber M (2017). Extensive proliferation of human cancer cells with ever-shorter telomeres. Cell Rep.

[36] Henson JD, Lau LM, Koch S, Martin La Rotta N, Dagg RA, Reddel RR (2017). The C-Circle Assay for alternative-lengthening-of-telomeres activity. Methods.

[37] Pickett HA, Cesare AJ, Johnston RL, Neumann AA, Reddel RR (2009). Control of telomere length by a trimming mechanism that involves generation of t-circles. EMBO J.

[38] Slatter T, Gifford-Garner J, Wiles A, Tan X, Chen YJ, MacFarlane M, Sullivan M, Royds J, Hung N (2010). Pilocytic astrocytomas have telomere-associated promyelocytic leukemia bodies without alternatively lengthened telomeres. Am J Pathol.

[39] Fogli A, Demattei MV, Corset L, Vaurs-Barriere C, Chautard E, Biau J, Kemeny JL, Godfraind C, Pereira B, Khalil T (2017). Detection of the alternative lengthening of telomeres pathway in malignant gliomas for improved molecular diagnosis. J Neurooncol.

[40] Zheng XH, Nie X, Fang Y, Zhang Z, Xiao Y, Mao Z, Liu H, Ren J, Wang F, Xia L (2017). A cisplatin derivative tetra-Pt(bpy) as an oncotherapeutic agent for targeting ALT cancer. J Natl Cancer Inst.

[41] Martinez AR, Kaul Z, Parvin JD, Groden J (2017). Differential requirements for DNA repair proteins in immortalized cell lines using alternative lengthening of telomere mechanisms. Genes Chromosomes Cancer.

[42] Lee M, Napier CE, Yang SF, Arthur JW, Reddel RR, Pickett HA (2017). Comparative analysis of whole genome sequencing-based telomere length measurement techniques. Methods.

[43] Farmery JHR, Smith ML, Diseases NB-R, Lynch AG (2018). Telomerecat: a ploidy-agnostic method for estimating telomere length from whole genome sequencing data. Sci Rep.

[44] Barthel FP, Wei W, Tang M, Martinez-Ledesma E, Hu X, Amin SB, Akdemir KC, Seth S, Song X, Wang Q (2017). Systematic analysis of telomere length and somatic alterations in 31 cancer types. Nat Genet.

[45] Conomos D, Stutz MD, Hills M, Neumann AA, Bryan TM, Reddel RR, Pickett HA (2012). Variant repeats are interspersed throughout the telomeres and recruit nuclear receptors in ALT cells. J Cell Biol.

[46] Varley H, Pickett HA, Foxon JL, Reddel RR, Royle NJ (2002). Molecular characterization of inter-telomere and intra-telomere mutations in human ALT cells. Nat Genet.

[47] Rosswog C, Bartenhagen C, Welte A, Kahlert Y, Hemstedt N, Lorenz W, Cartolano M, Ackermann S, Perner S, Vogel W (2021). Chromothripsis followed by circular recombination drives oncogene amplification in human cancer. Nat Genet.

[48] Feuerbach L, Sieverling L, Deeg KI, Ginsbach P, Hutter B, Buchhalter I, Northcott PA, Mughal SS, Chudasama P, Glimm H (2019). TelomereHunter - in silico estimation of telomere content and composition from cancer genomes. BMC Bioinformatics.

[49] Robinson JT, Thorvaldsdottir H, Winckler W, Guttman M, Lander ES, Getz G, Mesirov JP (2011). Integrative genomics viewer. Nat Biotechnol.

[50] Thorvaldsdottir H, Robinson JT, Mesirov JP (2013). Integrative Genomics Viewer (IGV): high-performance genomics data visualization and exploration. Brief Bioinform.

[51] Dobin A, Davis CA, Schlesinger F, Drenkow J, Zaleski C, Jha S, Batut P, Chaisson M, Gingeras TR (2013). STAR: ultrafast universal RNA-seq aligner. Bioinformatics.

[52] Cesare AJ, Heaphy CM, O'Sullivan RJ (2015). Visualization of telomere integrity and function in vitro and in vivo using immunofluorescence techniques. Curr Protoc Cytom.

[53] Lincz LF, Scorgie FE, Garg MB, Gilbert J, Sakoff JA (2020). A simplified method to calculate telomere length from Southern blot images of terminal restriction fragment lengths. Biotechniques.

