# Supplementary figures and images for: Reliable assessment of telomere maintenance mechanisms in neuroblastoma
Source: Cell Biosci. 2022 Sep 24;12:160. doi: 10.1186/s13578-022-00896-2 (PMC9508734; doi:10.1186/s13578-022-00896-2)

Supplementary Figure 1

A

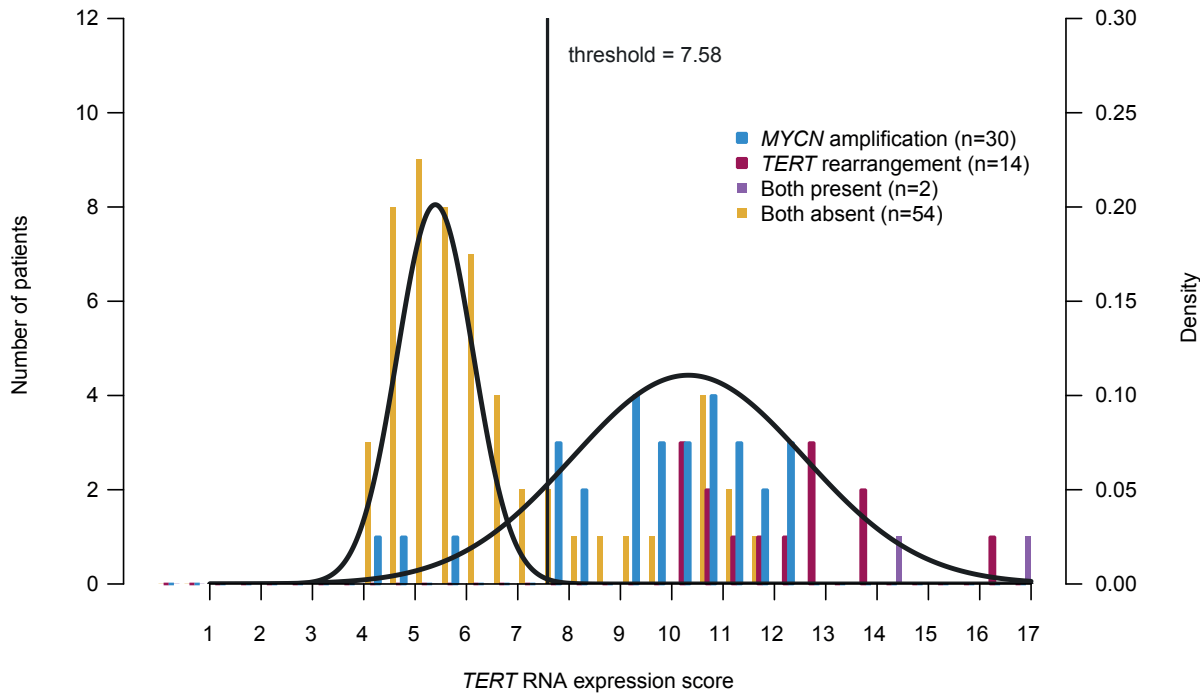

B

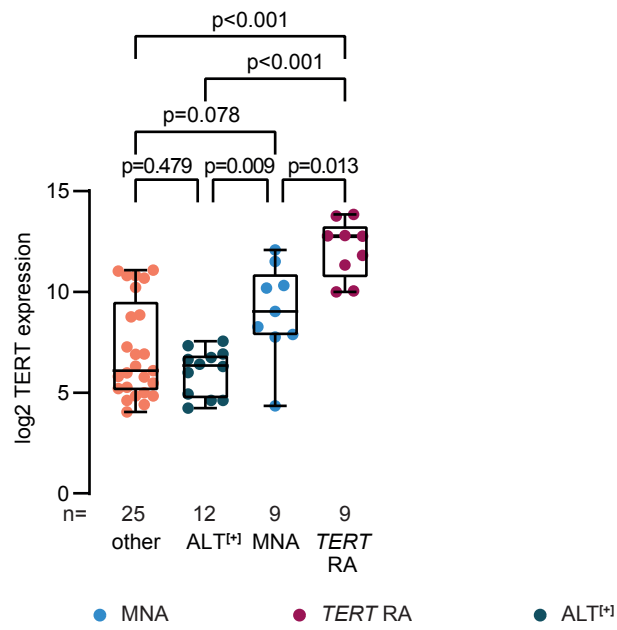

C

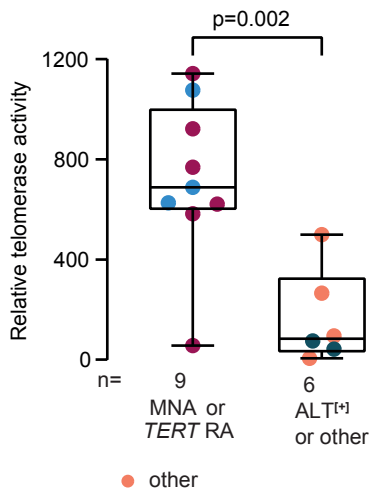

Supplement: Supplementary file 4 — Additional file 4: Figure S1. TERT expression and telomerase activity in neuroblastoma with activated telomere maintenance mechanism. (A) Distribution of TERT log2 expression values, determined by RNA sequencing, in neuroblastomas harboring TERT rearrangements, and/or MNA or none of these alterations. The threshold at 7.58 was defined as the lowest expression value having a posterior probability ≥95% to fall within the distribution on the right (i.e., the group of tumors with TERT/MYCN alteration). (B) Log2 TERT mRNA expression levels, dependent on telomere maintenance subgroup. ANOVA, Tukey’s multiple comparison test. n=55, RNA sequencing data was available for 64 cases, however ambiguous cases (n=7) as well as cases that show MNA and TERT RA in the same tumor (n=3) were excluded (see also Fig. 1B). Whiskers are limited to 1.5x interquartile range. (C) Relative telomerase activity dependent on underlying alteration, determined by TRAP assay. Unpaired t-test. n=15. Whiskers are limited to 1.5x interquartile range. [file 13578_2022_896_MOESM4_ESM.pdf]

Supplementary Figure 2

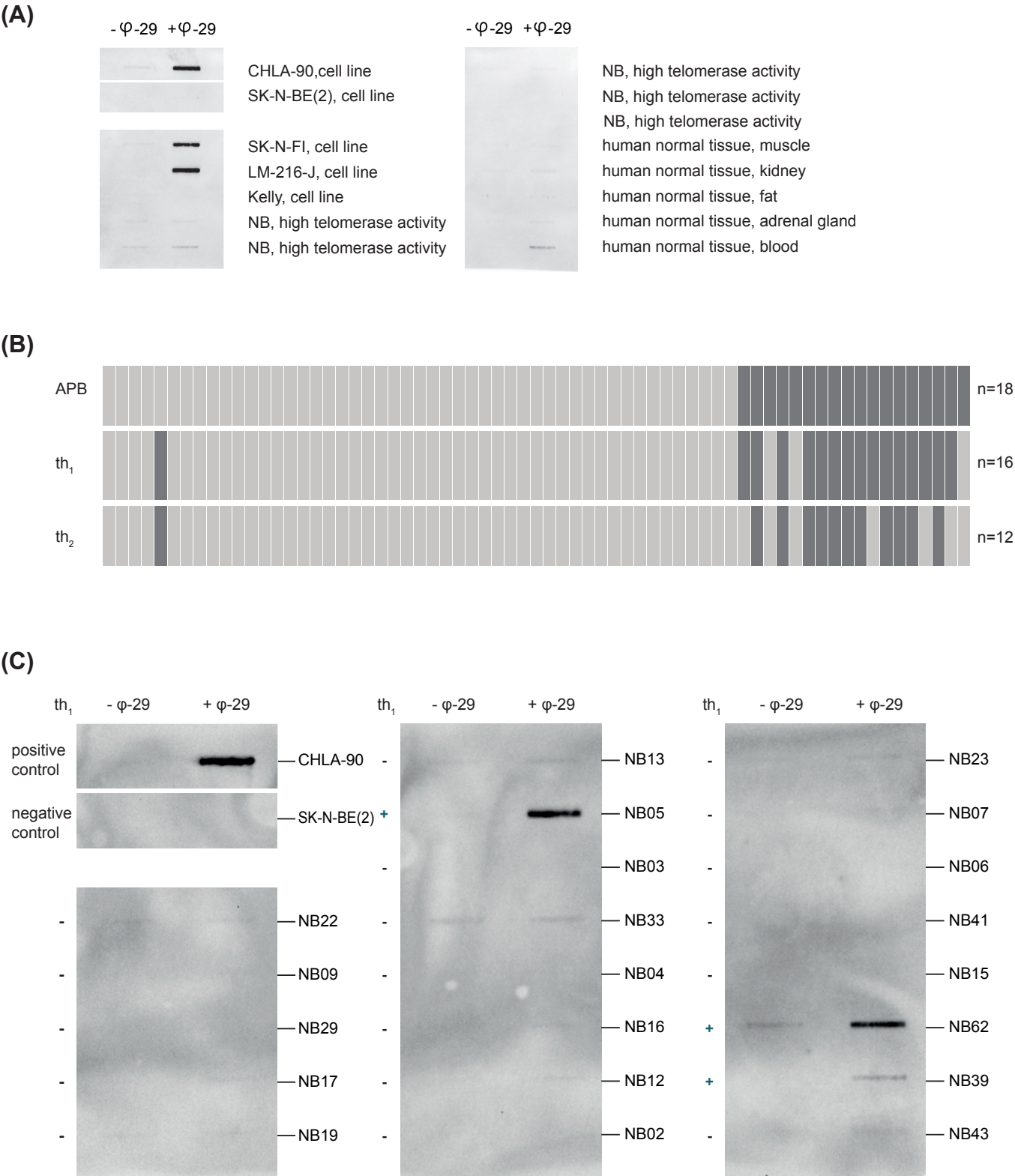

Supplement: Supplementary file 5 — Additional file 5: Figure S2. Dependency of C-circle assay on threshold and reference cell line. (A) Southern blot of C-circle assay of different cell lines (CHLA-90: ALT[+], SK-N-BE(2): TEL[+], SK-N-FI: ALT[+], LM-216-J: ALT[+]), normal human tissue and neuroblastoma samples with high telomerase activity (all ALT-negative). Left columns: sample without polymerase, right columns: samples with polymerase. (B) Neuroblastoma samples for which APB and C-circle assay was available (n=67). Different thresholds applied to the same samples reveal different results. (th1) C-circle signal intensity ≥5% relative to the signal of CHLA-90. (th2) C-circle signal intensity ≥20% relative to the signal of CHLA-90 and at least fourfold the area under the curve of polymerase-free dot-blot. Number of cases (n) classified as ALT-positive according to the respective threshold are indicated. (C) Representative image of southern blot of C-Circle assay of different cell lines and neuroblastoma samples. Left columns: sample without polymerase, right columns: samples with polymerase. ALT-positive cell line CHLA-90 and ALT-negative cell line SK-N-BE are depicted at the top, ALT status according to threshold th1 as indicated. [file 13578_2022_896_MOESM5_ESM.pdf]

Supplementary Figure 3

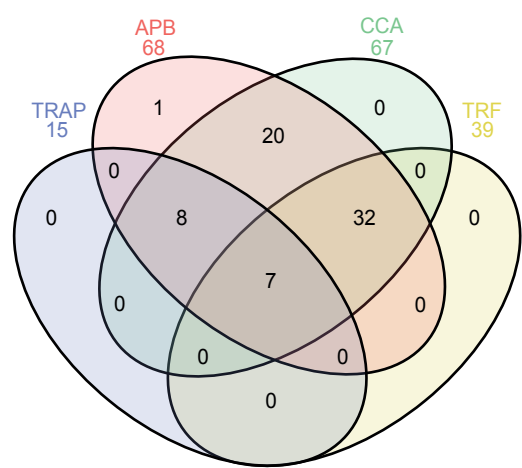

Supplement: Supplementary file 6 — Additional file 6: Figure S3. Venn diagram on availability of experimental data. [file 13578_2022_896_MOESM6_ESM.pdf]

Supplementary Figure 4

(A)

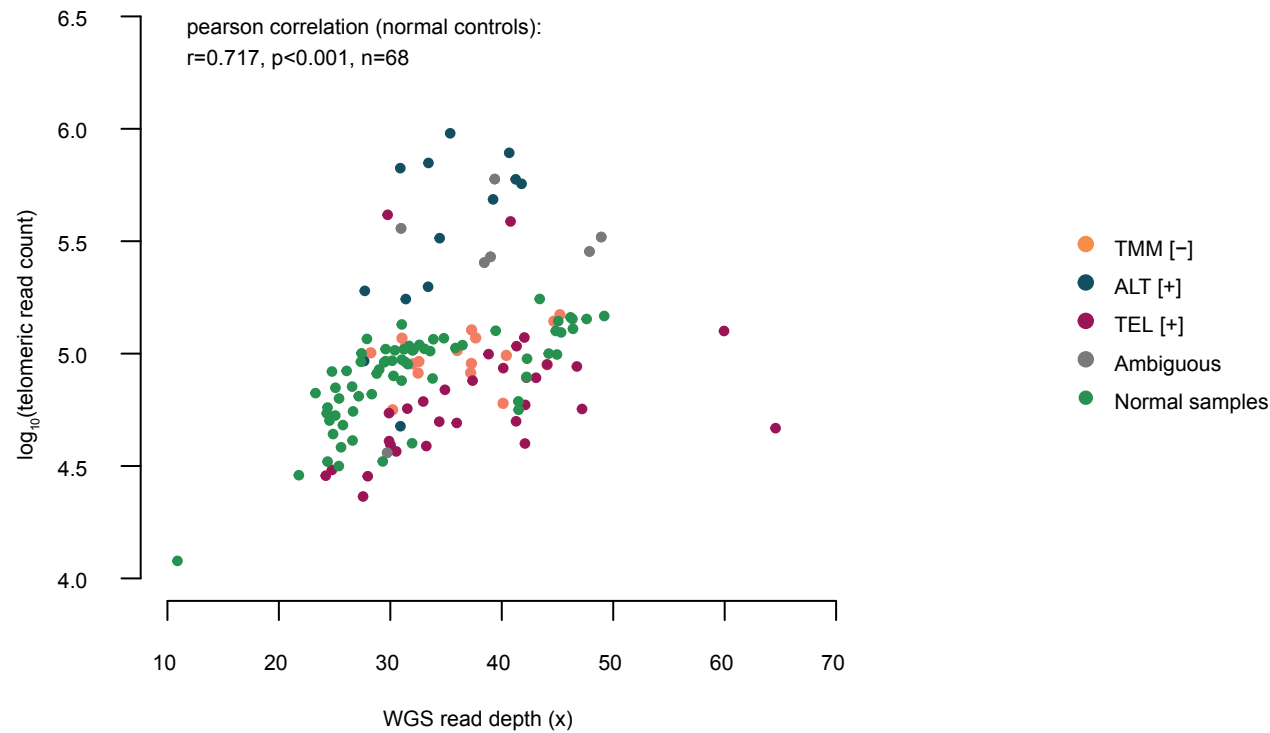

(B)

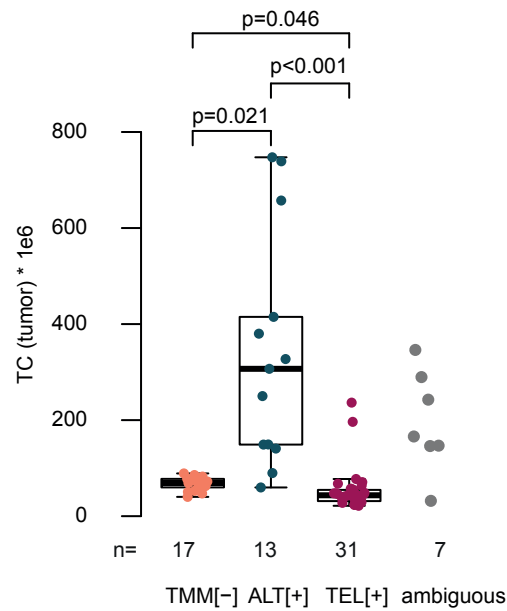

(C)

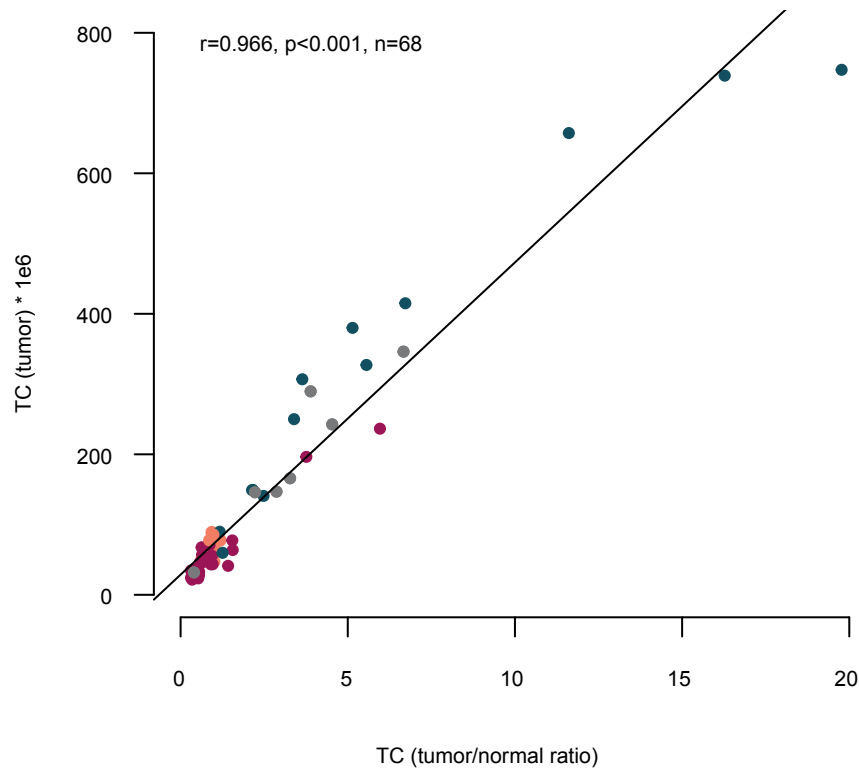

Supplement: Supplementary file 7 — Additional file 7: Figure S4. Detection of telomeric reads and calculation of telomere content on WGS data. (A) Number of detected telomeric reads, i.e., reads containing at least four t-type repeats, in tumor and normal WGS data in relation to the mean read depth. Pearson correlation displayed for normal controls only. (B) Normalized telomere content calculated from single tumor WGS data, dependent on telomere maintenance subgroups. Mean telomere content: TMM[-] 67.43, ALT[+] 339.33, TEL[+] 54.19. Kruskal-Wallis test and Dunn’s multiple comparison test, n=61, ambiguous cases were excluded from statistical analysis. Whiskers are limited to 1.5x interquartile range. (C) Correlation analysis of telomere content calculated from tumor/normal ratios and the respective single tumor samples. [file 13578_2022_896_MOESM7_ESM.pdf]

Supplementary Figure 5

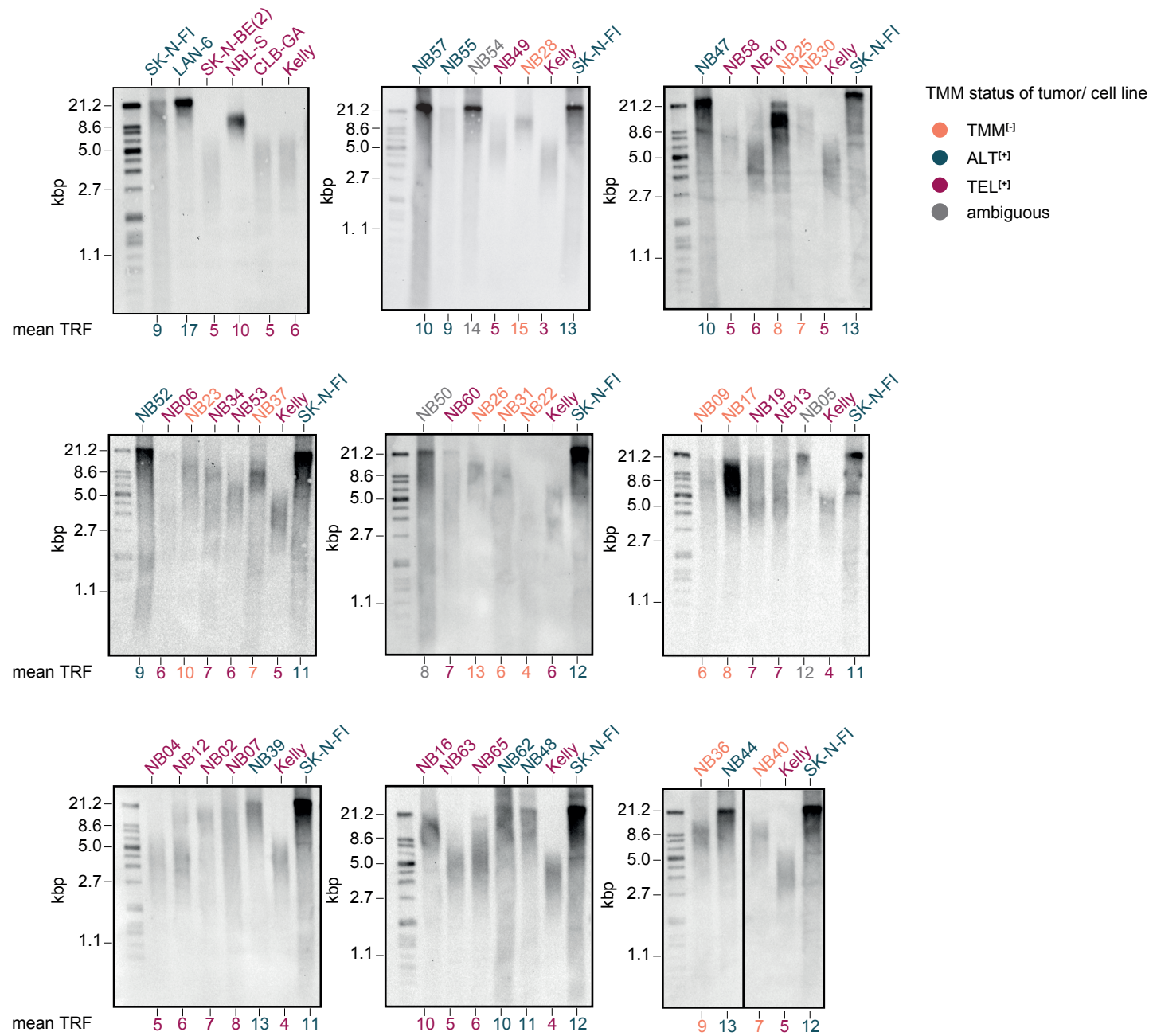

Supplement: Supplementary file 8 — Additional file 8: Figure S5. A distinct pattern of TRF southern blot analysis dependent on the TMM subgroup. Southern blot for telomere restriction fragment analyses of neuroblastoma samples and cell lines. ALT-positive cell line SK-N-FI and ALT-negative but telomerase-positive cell line Kelly were used on every blot with tumor samples as controls on every blot. [file 13578_2022_896_MOESM8_ESM.pdf]

Supplementary Figure 6

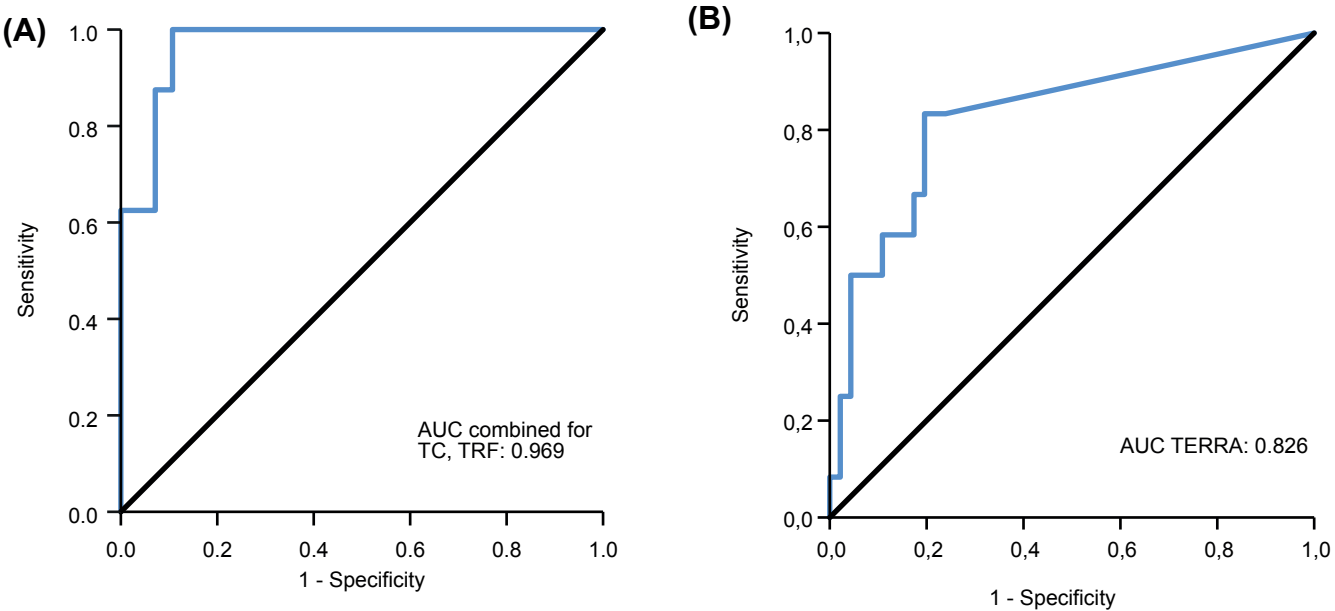

Supplement: Supplementary file 9 — Additional file 9: Figure S6. Features for prediction of TMM status in neuroblastoma. (A) Combined ROC for TC and TRF. After binary logistic regression and calculation of predicted probabilities, used for combined ROC, n=36. (B) ROC for normalized TERRA read count as a classifier for ALT, n=58. [file 13578_2022_896_MOESM9_ESM.pdf]

Supplementary Figure 7

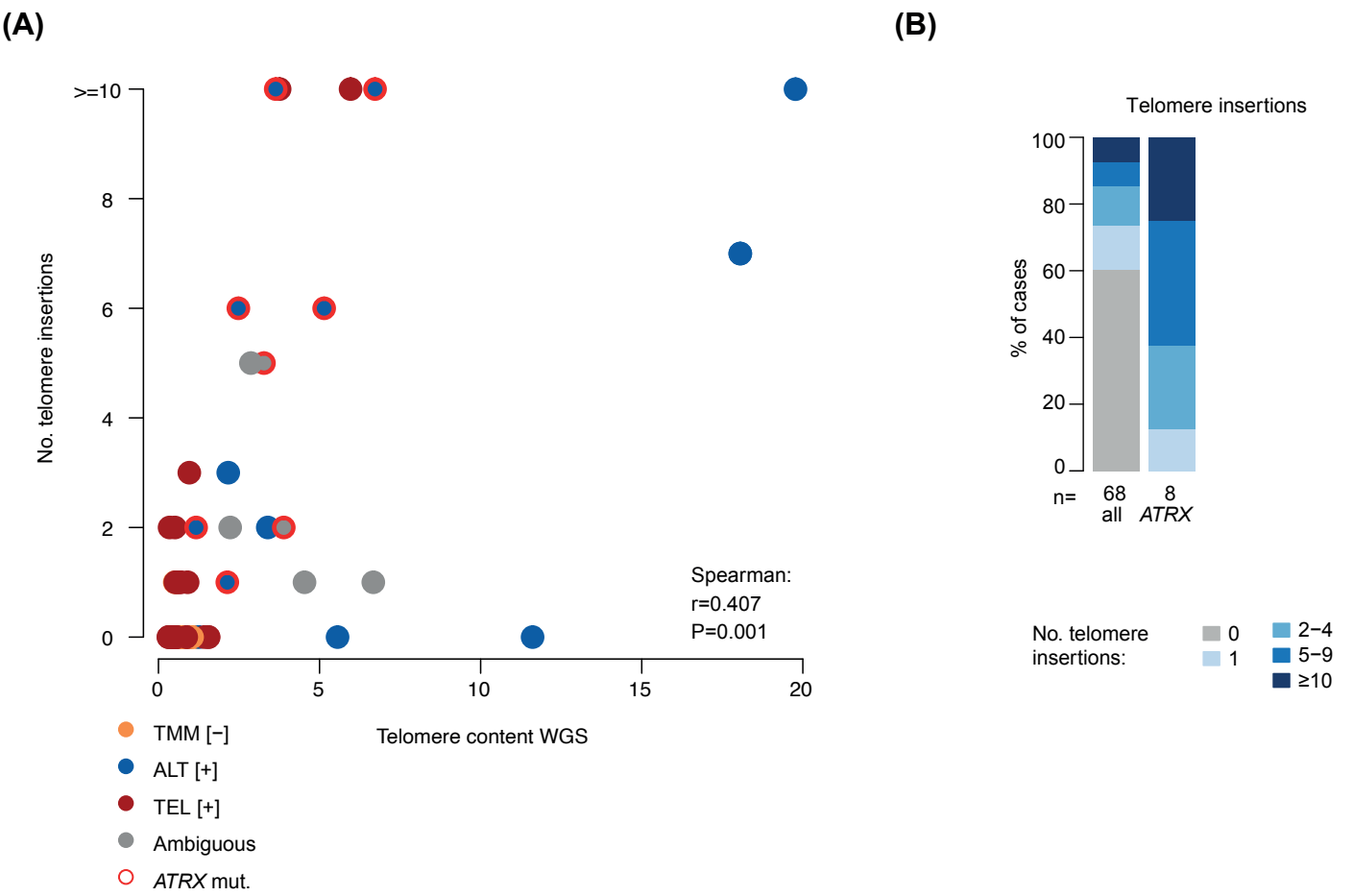

Supplement: Supplementary file 10 — Additional file 10: Figure S7. Telomere insertions in ALT-positive neuroblastoma. (A) Correlation analysis of numbers of telomere insertions into non-telomere regions and telomere content calculated on basis of WGS data, n=68. Ambiguous cases were excluded for spearman correlation. (B) Telomere insertions in the entire cohort and in subgroups of tumors with ATRX mutations. [file 13578_2022_896_MOESM10_ESM.pdf]

Supplementary Figure 8

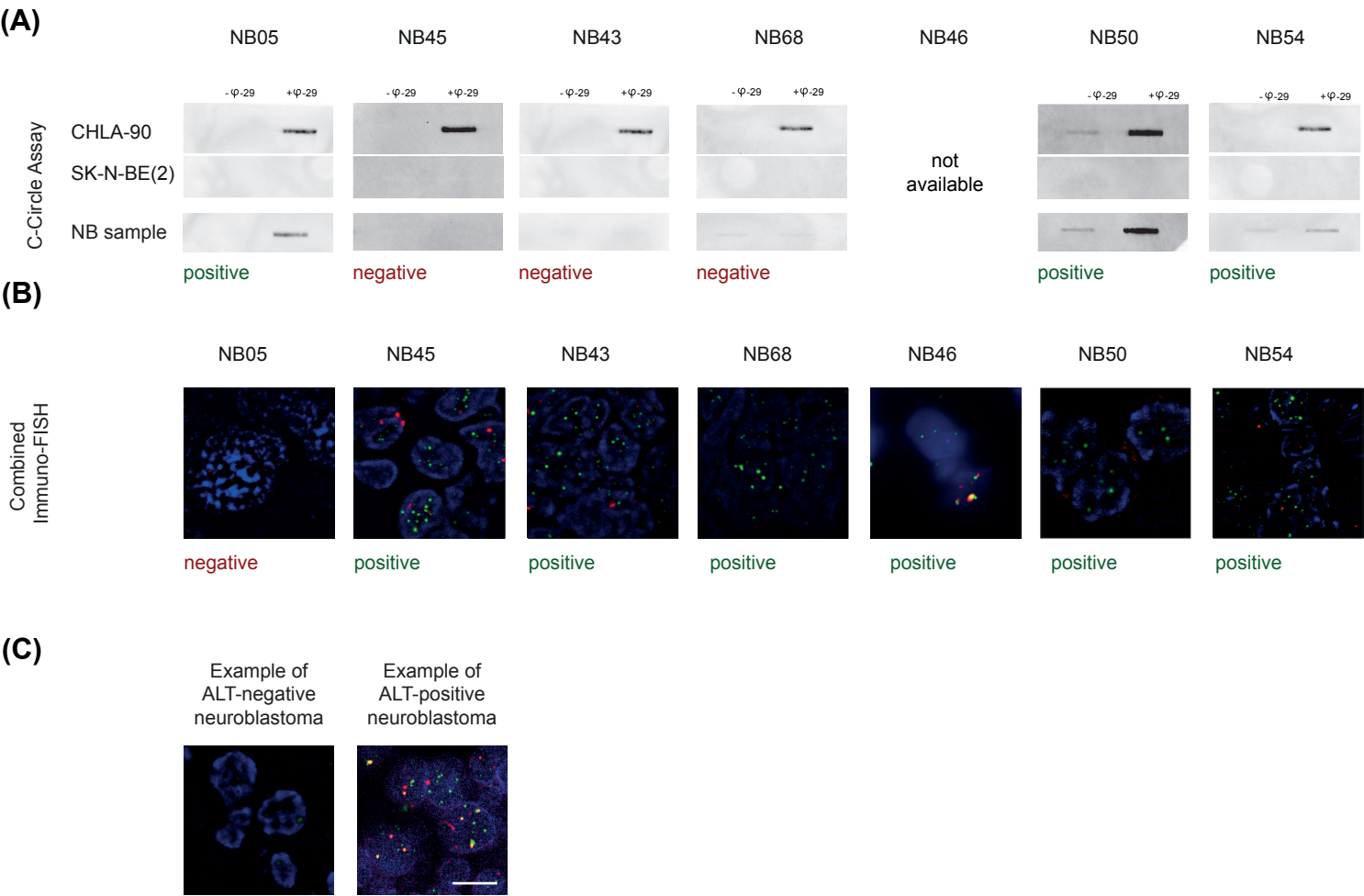

Supplement: Supplementary file 11 — Additional file 11: Figure S8. Raw data of CCA and combined Immunofluorescence/FISH of ambiguous neuroblastoma cases. (A) Images of CCA and (B) combined Immunofluorescence/FISH of ambiguous neuroblastoma samples with contrasting results as revealed by the two methods. Images of Immunofluorescence/FISH show ultrabright telomeric signals (green) and associated APBs (red) in ALT-positive cases, whereas the ALT-negative sample (NB05) does not show prominent telomeric signals and only subtle PML bodies. (C) Example of one unambiguous ALT-negative and one unambiguous ALT-positive neuroblastoma. [file 13578_2022_896_MOESM11_ESM.pdf]
